# Pilot-Scale Antioxidant Dipping of Herring (*Clupea harengus*) Co-products to Allow Their Upgrading to a High-Quality Mince for Food Production

**DOI:** 10.1021/acssuschemeng.2c07164

**Published:** 2023-03-13

**Authors:** Haizhou Wu, John Axelsson, Martin Kuhlin, Rikard Fristedt, Ingrid Undeland

**Affiliations:** † Department of Biology and Biological Engineering−Food and Nutrition Science, Chalmers University of Technology, SE 412 96 Gothenburg, Sweden; ‡ Sweden Pelagic AB, Hallgrens väg 1, SE 47431 Ellös, Sweden

**Keywords:** lipid oxidation, side streams, rest raw materials, rosemary extract, fish by-products, protein, biorefining

## Abstract

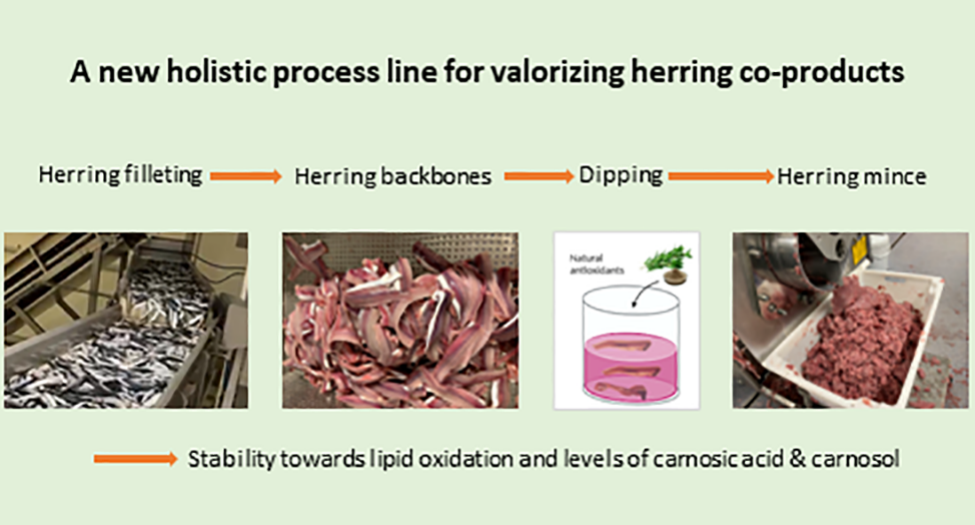

To enable production of high-quality mince from herring backbones, a scalable antioxidant strategy is needed due to the high susceptibility of herring muscle to lipid oxidation. We here measured the stabilizing effect of lab-/pilot-scale predipping of herring backbones (30–500 kg) in antioxidant solutions prior to production of mechanically separated mince (MSM). The antioxidants were (i) Duralox MANC, a mixture of rosemary extract, ascorbic acid, α-tocopherol, and citric acid, and (ii) rosemary extract with or without isoascorbic acid. Delivery of the key rosemary-derived antioxidant components carnosol and carnosic acid was monitored during the dipping process and ice/frozen storage. Predipping in 2% Duralox MANC gave MSM with 26.7–31.7 mg/kg carnosol + carnosic acid and extended the oxidation lag phase from <1 to 12 days during ice storage and from <1 to 6 months during frozen storage compared to control. Dipping in 0.2% rosemary extract with or without 0.5% isoascorbic acid solution gave MSM with 20.6–28.2 mg/kg carnosol + carnosic acid and extended the lag phase to 6 days and 9 months during ice and frozen storage, respectively. Our results confirmed, in pilot scale, that predipping herring coproducts in antioxidant solutions is a promising strategy to utilize these raw materials for, e.g., mince and burger production rather than for low value products as fish meal.

## Abstract 2

A novel holistic process line to valorize herring co-products to food rather than feed or waste is discussed.

## Introduction

Atlantic herring (*Clupea harengus*) is captured at an average of 2162 thousand tons per year and was ranked the fourth most landed fish worldwide between 1950 and 2017.^1^ Interestingly, this species yields a very low climate foot print with only 0.7 kg CO_2_ equivalents per kilogram, similar to soy beans.^2^ It also provides essential amino acids, minerals (e.g., iodine, selenium, calcium, and iron/heme-iron), and vitamins E, D, and B12, as well as the n-3 polyunsaturated fatty acids (PUFAS) eicosapentaenoic, docosahexaenoic, and docosapentaenoic acids (EPA, DPA, and DHA).^3^ Thus, herring is a very interesting contribution to the ongoing dietary protein shift in which consumers seek sustainable and nutritious alternatives to red meat. The general demand for seafood is also steadily increasing because of dietary recommendations and population growth; herring could here play a larger role than it does today. Salmonoid and white fish still dominate the global seafood consumption although a more diversified production and consumption would create better resilience in the seafood value chain.^1^ In many countries such as Sweden and Finland, large parts of the herring catches still go to feed in the form of fish meal/fish oil, which applies to both whole fish and coproducts. Regarding the latter, we recently reported that processing of round herring into fillets yields ∼60% coproducts (also called byproducts), comprising backbone, head, viscera, belly flap, and tail.^4^ Based on their high levels of residual muscle, the herring backbones have been identified as particularly promising for valorization into food products.^5^

A simple and low-cost technique to enhance the use of relatively clean fish coproduct parts is mechanical meat–bone separation.^5,6^ The mechanically separated muscle (MSM) has revealed great potential to be converted, e.g., into fish burgers.^7^ In MSM from herring backbones, the high levels of heme-proteins and PUFA however give rise to rapid lipid oxidation (i.e., rancidity), something which we recently detected already within 1 day on ice in the form of high peroxide value (PV) and TBA reactive substances (TBARS) levels.^8^ Indeed, fast oxidation limits the applicability of MSM within the food value chain and needs to be mitigated.

Direct addition of antioxidants into ground muscle tissue is a common method to prevent lipid oxidation in fish. However, a relatively long and intense mixing process is required to homogeneously disperse the antioxidants, especially if only small amounts are added.^9^ This process causes loss of endogenous muscle structure and exposes the main oxidation substrate, the phospholipid membranes,^10,11^ to pro-oxidative hemoglobin (Hb) and lipoxygenase, which stimulate the rate of lipid oxidation.^12^ Further to this, oxygen is dispersed into the system, unless the mixing is done under vacuum.

To avoid the limitations of direct antioxidant addition, we developed a lab-scale dipping technology in which the antioxidant solution could be efficiently recycled up to 10 times. Dipping mixed herring coproducts in 2% Duralox MANC (a mixture of rosemary extract, ascorbic acid, tocopherols, and citric acid) or in 0.2% rosemary extract with or without 0.5% isoascorbic acid considerably prolonged the oxidation lag phase from <1 day to >12 days during ice storage of the herring coproducts in minced or intact form.^13,14^ Also other investigators have successfully applied antioxidant dipping solutions on lab scale based on, e.g., basil extract,^15^ seaweed extract,^16^ and tilapia protein hydrolysates^17^ to stabilize different species of fish fillets.

However, to the best our knowledge, there are no published studies reporting on antioxidant dipping at pilot or full scale to inhibit lipid oxidation of fish. Similarly, knowledge is lacking about how much of the antioxidants are delivered from the solution to the fish material during different versions of dipping. The latter is of high importance to better understand underlying mechanisms of action and to comply with food regulations such as those controlled by EFSA or FDA. The aims of this study were to evaluate whether predipping herring backbones at pilot scale could prevent lipid oxidation of produced MSM during subsequent ice and frozen storage, as well as to monitor the delivery of key antioxidant components, e.g., carnosol and carnosic acid, to herring muscle during the dipping process.

## Materials and Methods

### Chemicals and Natural Antioxidants

Streptomycin sulfate, acetonitrile, acetic acid, sodium chloride, isoascorbic acid, trichloroacetic acid, 2-thiobarbituric acid, and ferrous sulfate were obtained from Sigma Chemical Co. (St. Louis, MO, USA). Duralox MANC-213 was purchased from Kalsec (Kalamazoo, MI, USA). Rosemary extracts (carnosol + carnosic acid ≥15%) were supplied by Hunan Shineway Enterprise (Changsha, China). All other chemicals used were American Chemical Society grade or better.

### Preparation of Herring Backbones

Herring (*Clupea harengus*) was caught off from Limfjord, Denmark, or Central North Sea and supplied by Sweden Pelagic AB in Ellösbetween October 2020 and April of 2022. The time between catching and filleting was less than 48 h. The filleting and sorting were done by a line from Baader (Model Baader 36, Nordisher Maschinenbau Rudolf Baader Gmbh, Lubeck, Germany), which was then rebuilt to allow sorting of herring filleting side streams into four fractions (backbone, intestine, tail, and head). The backbones were either subjected to meat–bone separation (BAADER 601, Lübeck, Germany) (±predipping in antioxidant solution) within <1 h on site at the herring process plant or directly transported to Chalmers University of Technology for lab-scale dipping. Arrival in Chalmers was within 4 h after filleting, and during transportation, the samples were covered by a plastic bag filled with ice.

### Pilot-Scale Dipping and Ice Storage of MSM

Dipping solutions with Duralox MANC and rosemary extract had tap water (10–12 °C) as a base and were prepared according to our previous studies.^13,14,18^ For dipping of herring backbones in the pilot scale, about 1000 L of antioxidant solution with 2% Duralox MANC or 0.2% rosemary extract was prepared, and MSM produced from predipped raw materials was regularly sampled after 1–10, 100–110, 200–210, 300–310, and 400–410 kg to monitor if the antioxidant potential of the solution was maintained. When dipping in 0.2% rosemary extract, sampling points for MSM were only 1–10, 100–110, and 200–210 kg due to a smaller raw material batch. About 25 g of MSM from each sampling point was then mixed with streptomycin sulfate (200 ppm) to inhibit bacterial growth,^19^ and the sample was transferred to screw-capped Erlenmeyer flasks where it was flattened out into a thin layer (∼5 mm) at the bottom followed by storage on ice.^20^ Subsamples were taken out regularly as described in our earlier study.^21^

### Lab-Scale Dipping and Frozen Storage of Backbones or MSM

Fresh herring backbones were immersed in prechilled (4 °C) solutions of 2% Duralox MANC or 0.2% rosemary extract + 0.5% isoascorbic acid (see above) for 30 s in a 1:5 ratio (3 kg of backbones/15 L of solution). The procedure was repeated 10 times using the same solution so that 30 kg was dipped in total. The samples were immediately sent to a meat–bone separator machine (BAADER 601, Lübeck, Germany) to produce MSM. About 50 g of MSM from each treatment was packed in Polynova plastic bags (89 mm × 114 mm, 50 micron), and air was expelled by manually flattening the packages into ∼5 mm. Then, the samples were stored at −20 °C for up to 9 months with space between all samples to allow equal access to air.

For storage of intact backbones, the same dipping method was used although the dipped backbones were now put directly in a single layer inside the Polynova plastic bags (229 mm × 324 mm, 50 micron). These samples were stored at −20 °C for up to 12 months in the same way as above. At each sampling point and for each treatment, at least three individual backbones were ground together in the frozen state in a Waring blender (LB20E* variable speed laboratory blender, 400 W, Waring Commercial, USA) at 6000 rpm. One gram of shredded backbone tissue was subsequently used to measure lipid oxidation.

### Analysis of Lipid Oxidation

Peroxide value (PV) and thiobarbituric acid-reactive substances (TBARS) were measured to monitor the lipid oxidation development in MSM and intact backbones during ice and frozen storage according to the Wu et al. method.^22^ A 1 g sample from the respective storage trial was mixed with 10 mL of chloroform/methanol (2:1) and homogenized with a polytron (T18 digital Ultra-Turrax, IKA, Germany) for 15 s at 12,000 rpm. The sample was then mixed with 3.08 mL of sodium chloride solution (0.5%) and vortexed for 30 s, followed by centrifugation at 2000*g* for 10 min.

The lower phase (chloroform) was collected for PV analysis according to Larsson et al. method.^20^ Briefly, 2.0 mL of the chloroform extract was mixed with 1.33 mL of ice-cold chloroform–methanol (1:1), and then, 33.4 μL of iron(II) chloride (18 mM) as well as ammonium thiocyanate (8.76 M) were added with 4 s vortexing between each addition. The sample was kept at room temperature for 20 min, and then, the absorbance was recorded at 500 nm with a spectrophotometer (Cary 60 UV–vis, Agilent technologies, Santa Clara, CA, USA). Cumene hydroperoxide was used to prepare the standard curve, and PV was expressed as μmol lipid hydroperoxide/kg of herring mince.

The upper phase (water–methanol) was used to determine TBARS according to Schmedes et al. method.^23^ A 2.5 mL water–methanol extract aliquot was mixed with 2.5 mL of TBA reagent (including 5.0% TBA and 0.5% TCA) in a 15 mL screw capped test tube. Samples were heated in a boiling water bath for 30 min, and then, the tubes were cooled in tap water for at least 20 min. The absorbance was recorded at 532 nm. The standard curve was prepared with 1,1,3,3-tetraethoxypropane, and results were expressed as μmol TBARS/kg of herring mince.

### Sample Preparation for Carnosol and Carnosic Acid Analysis

To monitor the entire antioxidant delivery process, we measured the content of carnosol and carnosic acid in crude antioxidants (Duralox MANC and rosemary extract), antioxidant dipping solutions, and MSM from predipped herring backbones. Different carnosol/carnosic acid extraction methods were used for the three types of samples because they contained different levels of carnosol and carnosic acid. For the crude antioxidants, Duralox MANC (0.2 g) and rosemary extract (0.1 g) were dissolved in 2 and 10 mL of methanol, respectively. For dipping solution samples, 10 mL was freeze-dried at −53 °C and 0.01 hPa pressure for 24 h by using a freeze-dryer (Heto LyoPro 3000, Heto/Holten A&S, Allerød, Denmark). The dried residue was then dissolved in 4 mL of methanol. The methanol mixtures from crude antioxidants or dipping solutions were then sonicated in a sonic cleaning bath cooled on ice for 20 min, followed by centrifugation (2000*g* for 3 min) at 4 °C. The supernatant was used for quantitative analysis.

For the MSM from predipped backbones, 50 g samples were freeze-dried at −53 °C and 0.01 hPa pressure for 24 h. The dried samples were ground to a powder using a coffee mill (2393 OBH Nordica, Stockholm, Sweden) for 30 s. The powder samples (1.0 g) were mixed with 15 mL of methanol and sonicated for 20 min. The mixture was centrifuged (2000*g* for 3 min) at 4 °C, and the supernatant was transferred to a clean tube. The residue was re-extracted in the same manner with 15 mL methanol. The extracts were then combined and centrifuged (2000*g* for 3 min) at 4 °C. Then, 16 mL of the supernatant was dried under nitrogen gas, and the residue was diluted in 1 mL methanol, followed by centrifugation (2000*g* for 3 min) at 4 °C. The supernatant was used for quantitative analysis.

#### Ultraperformance Liquid Chromatography (UPLC) Quantification

Carnosol and carnosic acid were quantitated with an UPLC system (Shimadzu Corporation, Kyoto, Japan) equipped with a C18 phase column (Acquity UPLC BEH C18, 15 cm, 1.7 μm, Waters, Milford, MA, USA). A sample volume of 4 μL was injected into the system, and chromatographic separation was performed with a 0.3 mL/min flow rate over 15 min. Eluents were (A) 1% acetic acid in Milli-Q water and (B) 1% acetic acid in acetonitrile. Eluent gradient conditions were 95% A and 5% B at 0 min, followed by a gradual decrease of A to 25% during 4 min and then to 20% at 9 min and to 5% at 11 min; this last ratio was maintained until 13 min and then set to 95% A to the end of the run (15 min). The temperature of the samples (SIL-40C XS Autosampler, Shimadzu) and column oven (CTO-40C, Shimadzu) were set at 5 and 50 °C, respectively. Detection was performed using a PDA UV detector at a scanning 190–500 nm wavelength. Carnosic acid (17108689, Selleck Chemicals, Houston, TX, USA) and carnosol (17205690, Selleck Chemicals, Houston, TX, USA) analytical standards (16–500 ppm) were used for method development, peak detection, and quantification. The UPLC chromatogram of carnosol and carnosic acid and standard curves are shown in Figure S2.

### Statistical Analysis

All statistical analysis was conducted with SPSS software (IBM SPSS Statistics Version 22, IBM Inc., Chicago, IL, USA). The results were reported as mean ± standard deviation (SD) (*n* ≥ 2). Duncan’s multiple range test was used to compare the means. Variance (ANOVA) was used to analyze the differences between treatments and/or storage points. The threshold for significance for all tests was set at *p* < 0.05.

## Results and Discussion

### Evaluation of Pilot-Scale Dipping to Inhibit Lipid Oxidation during Ice Storage of MSM from Backbones

**Figure 1:**
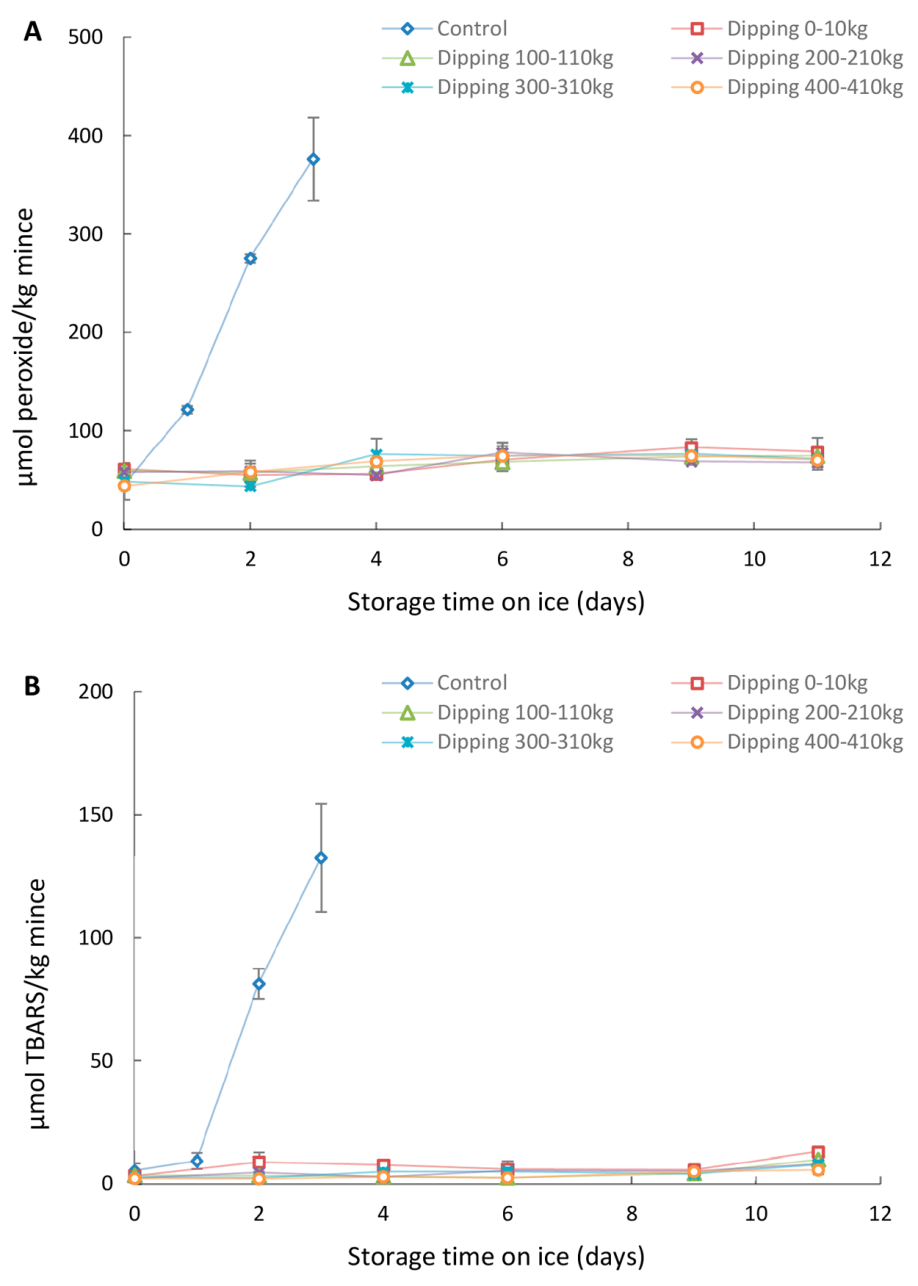
PV (A) and TBARS (B) development during ice storage of MSM from herring backbones with or without predipping in 2% Duralox MANC solution at pilot scale. Data are shown as mean values ± standard deviation (SD) (*n* = 2).

**Figure 2:**
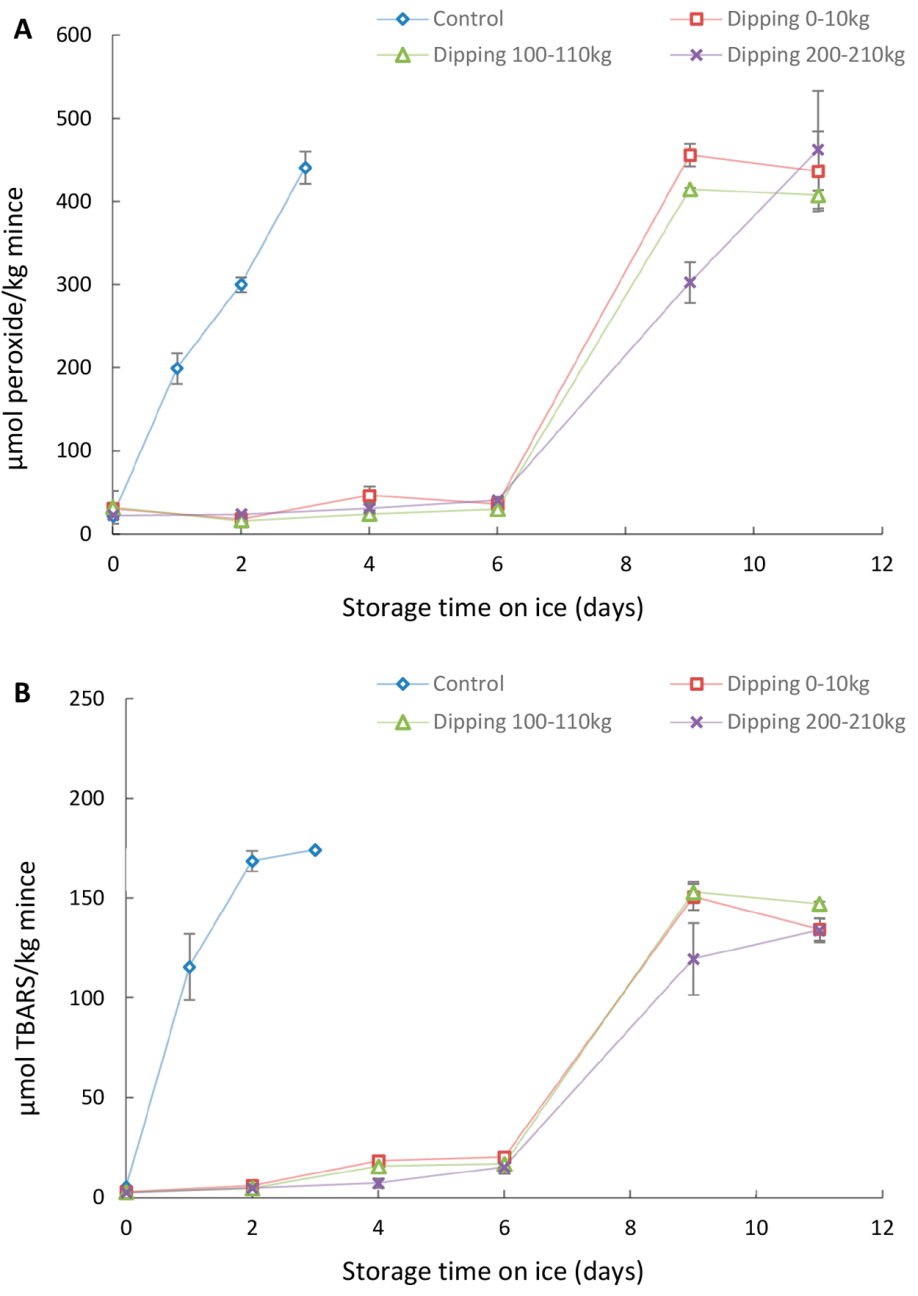
PV (A) and TBARS (B) development during ice storage of MSM from herring backbones with or without predipping in 0.2% rosemary extract solution at pilot scale. Data are shown as mean values ± standard deviation (SD) (*n* = 2).

In our previous lab-scale study (Figure S1), dipping mixed herring coproducts in 2% Duralox MANC or 0.2% rosemary extract with or without 0.5% isoascorbic acid prior to their mincing considerably increased the oxidation lag phase from <1 day to >12 days during ice storage.^13,14^ Even after reuse of the antioxidant solutions up to 10 times, lipid oxidation of the produced mince was completely inhibited during ice storage. In the present study, dipping of the backbone fraction derived from the herring coproducts was done in pilot scale, followed by MSM production. Figures 1 and 2 show the PV and TBARS values of MSM from backbones dipped with 2% Duralox MANC and 0.2% rosemary extract, respectively.

In both trials, PV and TBARS of the nondipped control samples had increased (*p* < 0.05) already after 1 day of storage revealing that herring backbone MSM is highly susceptible to lipid oxidation. This observation agreed with our previous findings for herring backbone minces^22^ and herring backbone MSM;^8^ that is, the lag phase for lipid oxidation was <1 day whether or not the backbones were nondipped or dipped in water or 0.9% NaCl. Herring MSM had a faster rate of lipid oxidation compared with minced herring fillets^20^ or MSM from salmon and cod.^8^ This can be attributed to a higher level of pro-oxidative Hb of herring MSM (35 μmol/kg mince) compared with herring fillet mince (14.5 μmol/kg mince),^22^ cod MSM (24 μmol/kg mince), and salmon MSM (14 μmol/kg mince).^8^ In addition, herring MSM also showed lower concentrations of α-tocopherol and ascorbic acid compared with salmon and cod MSM. On the basis of PV, hexanal, and TBARS, Cai et al.^24^ reported that Hb contributed to >90% of the total lipid oxidation in minced trout muscle during 9 days of 2 °C storage. Undeland et al.^11^ also reported that total Hb, but not total lipid level, controlled the lipid oxidation rate and intensity in a washed cod mince model.

Figure 2 shows that both PV and TBARS of MSM from backbones dipped in 0.2% rosemary extract did not increase significantly from day 0 to day 6. Similarly, lab-scale dipping of mixed herring coproducts in 0.2% rosemary extract showed effective inhibition of lipid oxidation during subsequent storage of the minced coproducts (Figure S1). Our results agreed with those of Karoui et al.^15^ who found that dipping in 1% (w/v) rosemary extract solution inhibited lipid oxidation based on measurement of TBARS of Atlantic mackerel (*Scomber scombrus*) fillets stored at 2 °C. The strong inhibitory effect of rosemary extract could be ascribed to carnosol and carnosic acid. Carnosol and carnosic acid account for over 90% of the antioxidant properties of rosemary extract.^25^ Both compounds contain phenolic rings with a high degree of methylation and hydroxylation which could donate electrons to neutralize reactive oxygen species and free radicals as free radical scavenging.^26^ In addition, the ability of carnosol and carnosic acid to reduce metHb to oxyHb may be an important mechanism that prevents the pro-oxidant activity of Hb.^27^ An interesting observation in this trial was that at day 9 lipid oxidation levels were significantly (*p* < 0.05) lower in MSM from the last dipping cycle than the two first ones. Possibly, the increase of muscle juices from the backbones in the dipping solution provided another array of antioxidants. Our earlier work has revealed a very strong antioxidant capacity of herring muscle press juice and fish blood plasma toward Hb-mediated membrane lipid oxidation; candidate compounds have been, e.g., ascorbic acid and uric acid.^28^

Compared with 0.2% rosemary extract (inhibiting 6 days), 2% Duralox MANC had a greater ability to prevent the development of PV and TBARS in MSM (inhibiting 11 days) (Figure 1). The additional antioxidants in Duralox MANC, i.e., tocopherol, ascorbic acid, and citric acid, provide additional radical scavenging, reducing and chelating properties thus interfering with a wider range of oxidation mechanisms. As an example, we previously found that Duralox MANC prevented auto-oxidation and hemin loss of herring Hb.^13^ It is also possible that the three additional compounds of Duralox MANC act synergistically with carnosol and carnosic acid from the rosemary extract. In an earlier study, a combination of rosemary extract and α-tocopherol (0.02% + 0.05%) showed a higher antioxidant activity in frozen sardine muscle and delayed the onset of lipid oxidation 5 more days than either rosemary extract or α-tocopherol alone.^29^ Similarly, Hraš et al.^30^ reported that, in sunflower oil stored at 60 °C, a mixture of rosemary extract and citric acid was a more effective oxidation inhibitor compared with rosemary extract alone. Thus, a variety of inhibitory effects may explain the high effectiveness of Duralox MANC in preventing lipid oxidation of herring MSM.

### Effect of Dipping Backbones on MSM Stability during Frozen Storage

**Figure 3:**
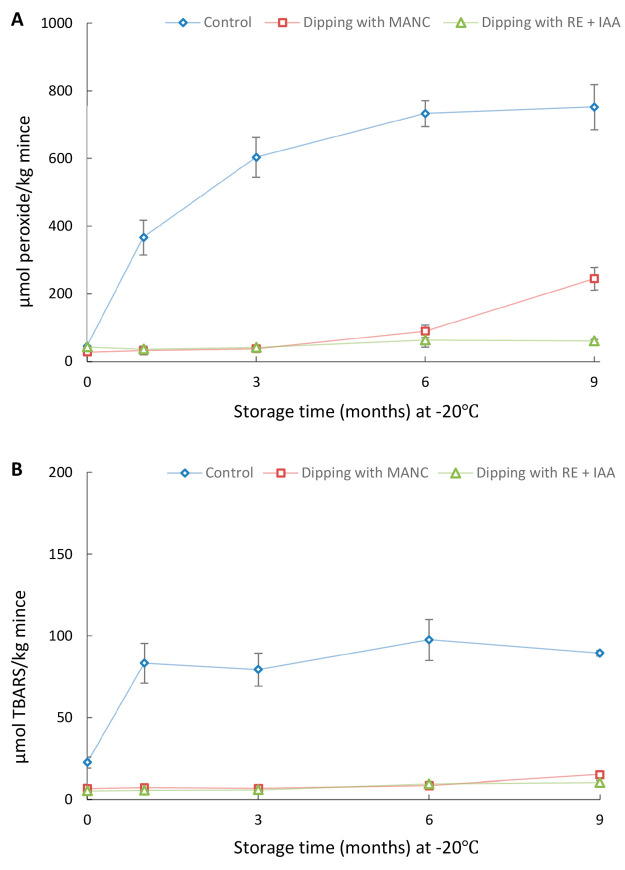
PV (A) and TBARS (B) development during frozen storage of MSM derived from herring backbones predipped in 2% Duralox MANC or 0.2% rosemary extract + 0.5% isoascorbic acid at lab scale. The ratio of herring backbones to solution was 1:5 (weight:volume), and the dipping solutions were reused 10 times. Predipped backbones from all 10 batches were mixed together prior to mechanical meat–bone separation. Data are shown as mean values ± standard deviation (SD) (*n* = 2).

Figure 3 shows the development of PV and TBARS of MSM from predipped or nondipped herring backbones during frozen storage. In this trial, dipping was done in lab scale, and the dipping solution was reused up to 10 times. Between 0 and 1 month, PV of MSM from nondipped backbones displayed a significant increase from 45.1 to 366.7 μmol/kg, and TBARS increased from 22.7 to 83.4 μmol/kg. These results indicated that herring backbone MSM is susceptible to lipid oxidation even when stored at −20 °C, a situation that challenges subsequent introduction into new herring products. Similarly, our previous study reported a rapid increase in PV and TBARS of herring backbone MSM during two months of frozen storage (−20 °C).^8^ Although low temperature in general reduces chemical reaction rates, including that for Hb-mediated lipid oxidation,^10^ certain freezing-induced changes of the fish muscle structure could facilitate lipid oxidation. For example, Jia et al.^31^ surmised that freezing could cause dehydration, increase the exposure of lipids to oxygen on the surface of the muscle tissue, and damage muscle cell membranes by the formation of ice crystals. Further to this, reactants can become concentrated in the unfrozen pools of water in the muscle, especially after slow freezing as was used here.^32^ Thus, the rate of lipid oxidation can still be relatively extensive under conventional frozen storage temperatures (−18 to 25 °C).

Figure 3 shows that predipping the backbones in 0.2% rosemary extract + 0.5% isoascorbic acid completely prevented the increase of PV and TBARS of produced MSM for 9 months of frozen storage, while predipping in 2% Duralox MANC completely inhibited formation of PV and TBARS for 6 months (Figure 3A) and 9 months (Figure 3B), respectively. In addition, this lab-scale trial showed that 15 L of antioxidant dipping solution could stabilize 30 kg of backbones against lipid oxidation as the solution could be reused up to 10 times. Based on these data, we predict that 0.5 ton of dipping solution per ton backbones would be enough also in a scaled-up scenario to stabilize produced MSM for 6–9 months at −20 °C.

**Figure 4:**
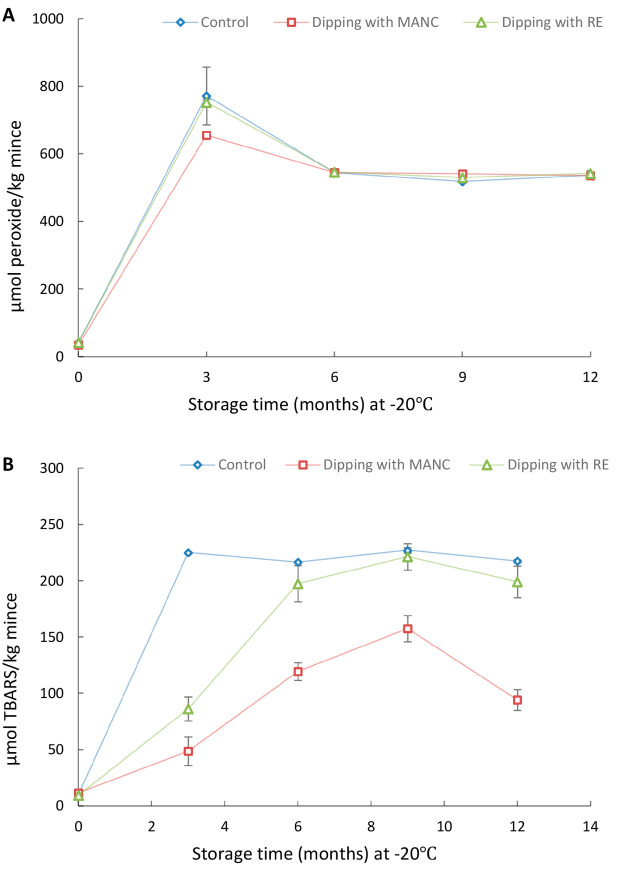
PV (A) and TBARS (B) development during frozen storage of intact herring backbones predipped in 2% Duralox MANC or 0.2% rosemary extract at lab scale. The ratio of herring backbones to solution was 1:5 (weight/volume) during the dipping. Data are shown as mean values ± standard deviation (SD) (*n* = 2).

The fish industry often suffers from seasonality and from lower capacity in side stream valorization processes than in the main production steps, e.g., the filleting lines.^8^ Freezing the side streams for later valorization could however be a route to even out the production peaks. Here, we investigated how predipping herring backbones affected lipid oxidation when they were frozen stored in the intact state. Figure 4 shows a significant increase in PV and TBARS of nondipped herring backbones already at 3 months compared with 0 months. Thus, just as MSM, intact herring backbones were highly susceptible to lipid oxidation during frozen storage. Interestingly, the intact herring backbones even showed significantly (*p* < 0.05) higher PV (752.9 > 603.7 μmol/kg) and TBARS (224.9 > 79.6 μmol/kg) compared with MSM at 3 months. The higher values were possibly attributed to a significantly (*p* < 0.05) higher Hb concentration in backbones compared with MSM, according to our recent study 43 vs 35 μmol/kg.^8^ Some of this Hb was also surface oriented. Another reason could be a higher surface-to-volume ratio of the backbones compared to the mince during frozen storage. Earlier research showed how lipid oxidation in minced herring was extremely surface oriented during frozen storage.^33^

Predipping the backbones in 2% Duralox MANC or 0.2% rosemary extract did not inhibit PV development in the intact backbones during storage at −20 °C, apart from a small but significant reduction at month 3 for Duralox MANC. For TBARS, predipping yielded significantly lower values after 3 months (Duralox MANC and rosemary extract), and after 6–12 months (Duralox MANC), compared to the control. This points at a capacity of Duralox MANC to prevent lipid hydroperoxide breakdown into carbonyls. However, backbones treated with both Duralox MANC or rosemary extract had both developed significantly higher TBARS values already after 3 months compared with month 0 (Figure 4B). This finding was different from our earlier storage trial with intact herring coproducts at 4 °C which showed that the same type of antioxidant predipping effectively inhibited both PV and TBARS.^14^ The failure of predipping to completely inhibit lipid oxidation of intact backbones during frozen storage could be attributed to damage of the antioxidant film formed on the backbone surface by migration, diffusion, and/or sublimation of ice from tissue.^31^ This would most likely be mitigated by vacuum packaging or other modes of tight packaging. In a scenario where backbones are predipped and then converted to MSM, the antioxidants on the surface will efficiently be mixed into the material and yield a sample less sensitive to film damage.

### Delivery of Carnosol and Carnosic Acid during Dipping

From a legislation perspective, and also to better understand the mechanisms by which predipping protects herring coproducts against oxidation, it is important to measure the concentrations of antioxidant components migrating from the dipping solution to the intermediate raw material and further to the MSM. We therefore monitored the levels of carnosol and carnosic acid in the crude antioxidants used, the dipping solution, the dipped backbone, and the MSM. Figure S3 shows that the contents of carnosol, carnosic acid, and carnosol + carnosic acid were 0.13, 0.73, and 0.86 g/100 g, respectively, in the Duralox MANC. For rosemary extract, the corresponding contents were 2.5, 13.9, and 16.4 g/100 g. Thus, the carnosol + carnosic acid was about 20-fold greater in the rosemary extract compared to in the Duralox MANC. The carnosol + carnosic acid amounts measured agreed with the values claimed by the suppliers regarding Duralox MANC (0.8%–0.9%) and rosemary extract (≥15%).

**Figure 5:**
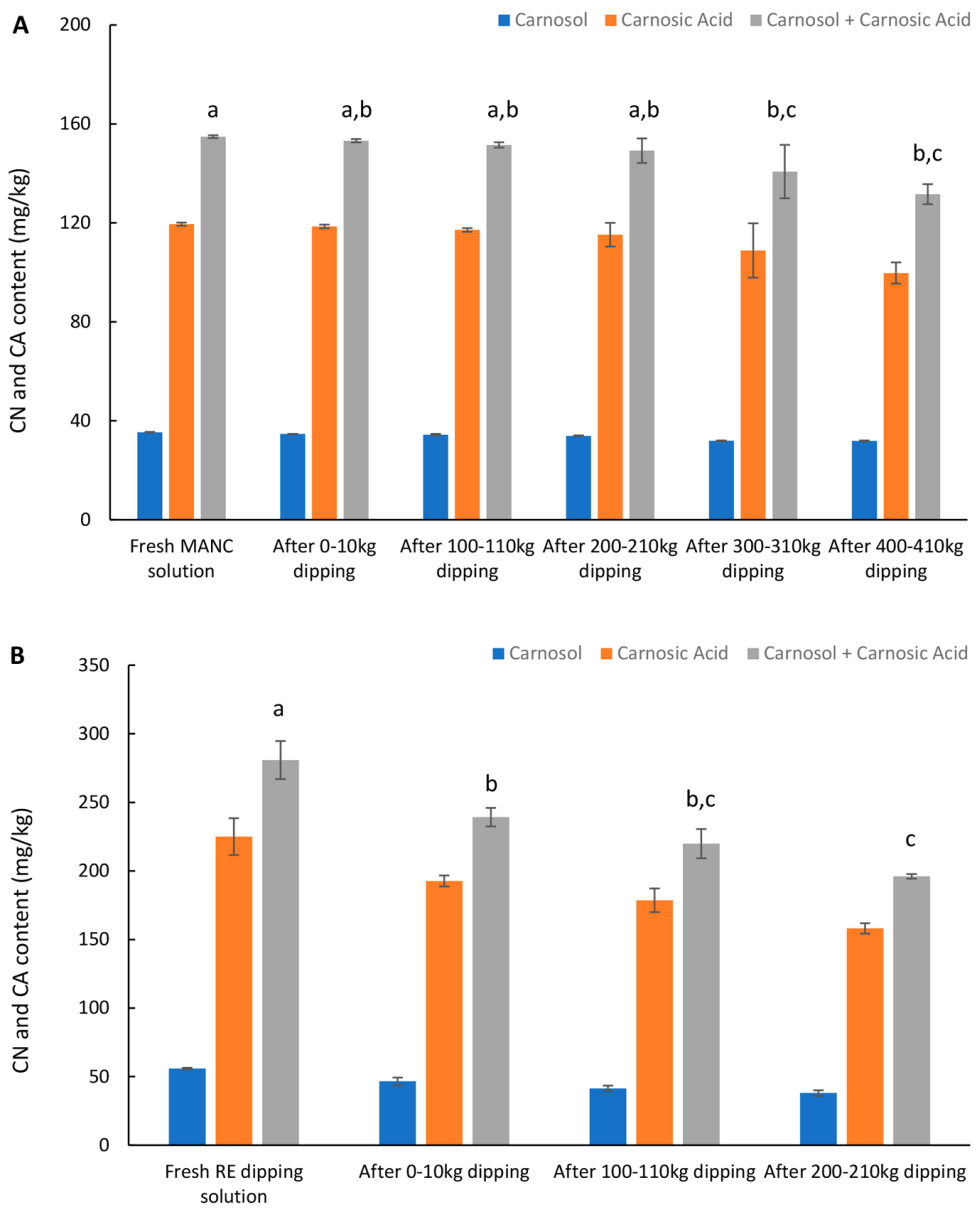
Content of carnosol and carnosic acid in the 2% Duralox MANC (A) and 0.2% rosemary extract (B) dipping solutions throughout their use for in total 500 and 250 kg of backbones, respectively. Data are shown as mean values ± standard deviation (SD) (*n* = 2).

The contents of carnosol and carnosic acid in the prepared solutions with 2% Duralox MANC or 0.2% rosemary extract, before, and after dipping in pilot scale, are shown in Figure 5. Carnosol, carnosic acid, and carnosol + carnosic acid were present at 35.4, 119.5, and 154.9 mg/kg in the initial Duralox MANC dipping solution, and at 55.9, 225.0, and 280.8 mg/kg in the rosemary extract dipping solution. Theoretical values based on the carnosol + carnosic acid levels in the crude Duralox MANC and rosemary extract combined with the dilution factors should have been 172 and 327 mg/kg in 2% Duralox MANC and 0.2% rosemary extract solution, respectively. Thus, we calculated that the Duralox MANC and rosemary extract dissolved to 90% and 85%, respectively, in the water. That Duralox MANC dissolved slightly better than rosemary extract may result in greater antioxidant availability to the dipped tissue. Figure 5A shows that the content of carnosol + carnosic acid in the 2% Duralox MANC solution after dipping 210 kg of herring backbones was not significantly (*p* > 0.05) different from the fresh Duralox MANC solution. However, the carnosol + carnosic acid content in the solution was slightly, yet significantly, lower (*p* < 0.05) than the fresh solution after dipping 310 and 410 kg of backbones (140.7 and 131.6 vs 154.6 mg/kg; Figure 5A). Regarding the rosemary extract solution (Figure 5B), the content of carnosol + carnosic acid was lower (*p* < 0.05) already after dipping 10 kg of backbones compared with the fresh solution. Throughout the entire dipping of 210 kg of backbones, the content of carnosol + carnosic acid decreased from 280.8 to 196.1 mg/kg. The sharp drop in carnosol + carnosic acid of the rosemary extract dipping solution could be attributed to muscle juice, blood, lipids, and/or other residues from the fish tissue being destroyed or coagulation of the antioxidant emulsified droplets, thereby reducing their dispersion. That Duralox MANC was less sensitive to such phenomenon could be due to its higher polarity.

**Figure 6:**
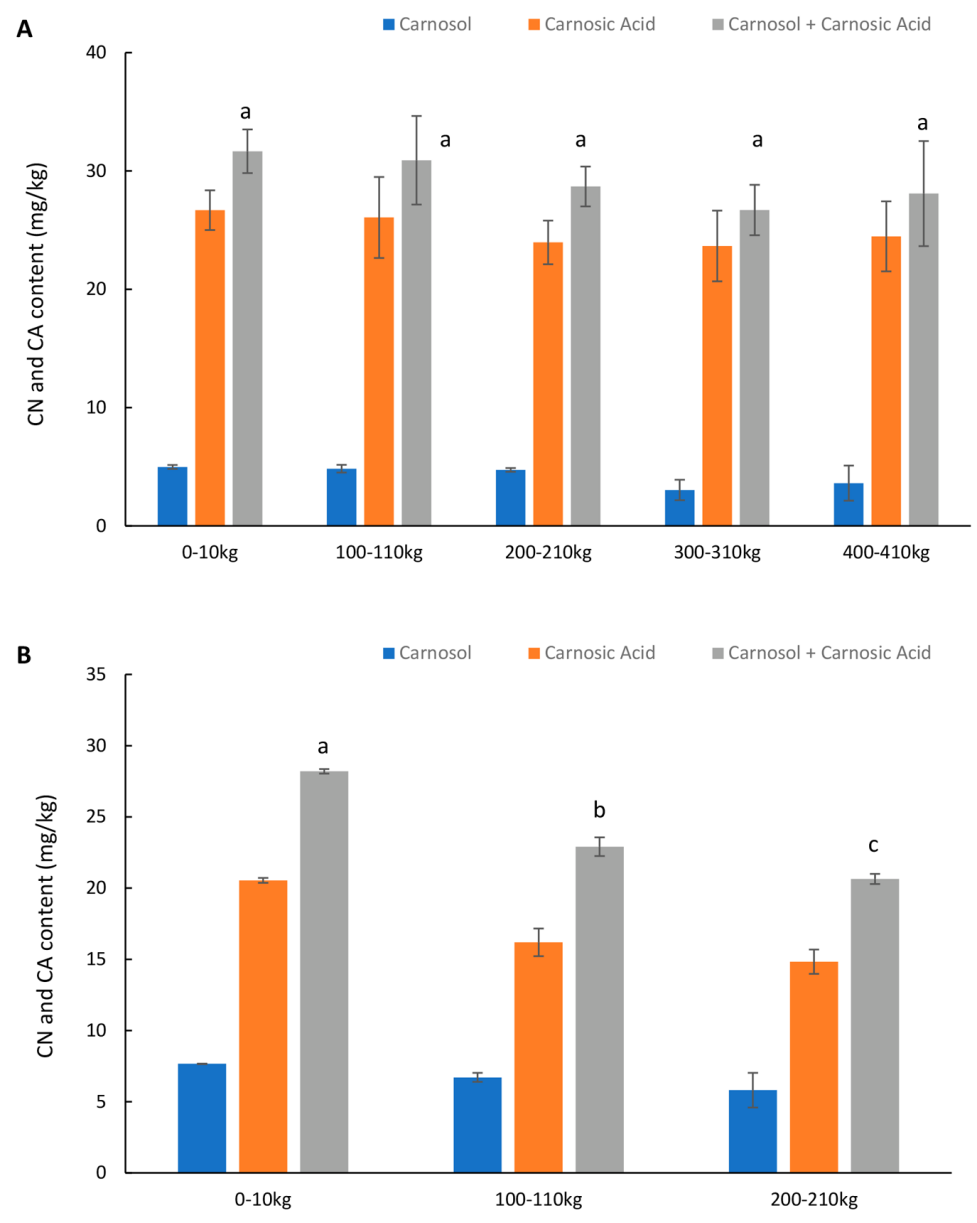
Content of carnosol and carnosic acid in MSM derived from backbones predipped in 2% Duralox MANC (A) or 0.2% rosemary extract (B) solution in pilot scale. Data are shown as mean values ± standard deviation (SD) (*n* = 2).

Figure 6 shows the contents of carnosol, carnosic acid, and carnosol + carnosic acid of MSM from backbones predipped in Duralox MANC and rosemary extract solution in pilot scale. The content of carnosol + carnosic acid of MSM derived from Duralox MANC predipping did not reveal any significant differences between the various sampling points, which ranged between 26.7 and 31.7 mg/kg MSM (Figure 6A). MSM derived from rosemary extract predipping however showed significant reductions in carnosol + carnosic acid throughout the dipping cycles, 28.2 mg/kg (0–10 kg) > 22.9 mg/kg (100–110 kg) > 20.6 mg/kg (200–210 kg) (Figure 6B). First, this reveals that carnosol and carnosic acid levels were within the rosemary extract (E 392) levels set as safe by the European Food Safety Authority (EFSA) in processed fish and fishery products (150 mg carnosol and carnosic acid/kg).^34^ Second, these results together with the results of Figure 2 indicate that ∼20 mg carnosol + carnosic acid/kg MSM was enough to inhibit lipid oxidation for up to 6 days on ice (Figure 2B) when production was done under pilot-scale conditions, but that higher carnosol + carnosic acid levels (≥26.7 mg/kg MSM) were needed to get an oxidation lag phase of 11 days (Figure 2A). This agrees with Hernández-Hernández et al.^35^ who reported that addition of rosemary extract to a final concentration of 22.5 mg carnosol + carnosic acid/kg patties could inhibit lipid oxidation of raw and cooked ground buffalo meat patties and chicken patties on ice for up to 5 days. It should however be stressed that additional trials are needed to confirm whether the ∼7 mg extra carnosol + carnosic acid/kg of MSM fully explains the 5 extra days gained in oxidation lag phase with Duralox MANC compared to rosemary extract. Indeed, the presence of tocopherol, ascorbic acid, and citric acid in the MSM most likely also contributed to the higher stability.

It was interesting to note that the crude Duralox MANC solution had a lower content of carnoso l+ carnosic acid than the crude rosemary extract, whereas MSM derived from backbones dipped in Duralox MANC had a higher carnosol + carnosic acid level compared with MSM derived from rosemary extract dipping. This difference could be attributed to differences in emulsion droplet sizes and stability, which may affect the delivery of carnosol + carnosic acid to the herring backbone.^36^ Future work should focus on the physical stabilities of the emulsified carnosol and carnosic acid in the dipping solution, e.g., with respect to used surfactant, droplet size, droplet stability, and viscosity of the continuous phase.

## Conclusions

MSM derived from herring backbones was found to be highly susceptible to lipid oxidization with a lag phase of <1 day on ice and <1 month at −20 °C. When the herring backbones were predipped in 2% Duralox MANC on a pilot scale prior to mechanical meat–bone separation, the oxidation lag phase of the MSM was however extended to 12 days on ice and 6 months at −20 °C. In a similar manner, predipping herring backbones in 0.2% rosemary extract with or without 0.5% isoascorbic acid extended the oxidation lag phase of MSM to 6 days on ice and 9 months at −20 °C. This indicated that the lipid oxidation-inhibiting effect from dipping that we have previously documented on lab scale was stable during a transformation to pilot scale. Based on the results, we could also predict that only 0.5 ton of dipping solution would be needed per ton of backbones to stabilize MSM in a further scaled-up scenario. The content of carnosol and carnosic acid in the MSM produced from pre-dipped backbones was within the limit recommended by EFSA and paves the way for a new scalable technology to valorize herring coproducts to food rather than feed.

## Supporting Information Available

Information as mentioned in the text (PDF)
