# Supplementary material for: Pilot-Scale Antioxidant Dipping of Herring (Clupea harengus) Co-products to Allow Their Upgrading to a High-Quality Mince for Food Production
Source: ACS Sustain Chem Eng. 2023 Mar 13;11(12):4727–37. doi: 10.1021/acssuschemeng.2c07164 (PMC10064803; doi:10.1021/acssuschemeng.2c07164)

Supporting Information for

Pilot-scale antioxidant dipping of herring (*Clupea harengus*) by-products to allow their upgrading to a high-quality mince for food production

Haizhou Wu [1,*], John Axelsson[1], Rikard Fristedt[1], Martin Kuhlin[2], Ingrid Undeland[1]

[1]Department of Biology and Biological Engineering–Food and Nutrition Science, Chalmers University of Technology, SE 412 96, Gothenburg, Sweden

[2]Pelagic Sweden AB, Hallgrens väg 1A, 474 31 Ellös, Sweden

*Corresponding author: Haizhou Wu

Department of Biology and Biological Engineering–Food and Nutrition Science

Chalmers University of Technology, SE 412 96, Gothenburg, Sweden

Tel: +46 317723845

Email: haizhou@chalmers.se; wuhaizhouing@gmail.com

Total number of pages: 4

Total number tables: 0

Total number of figures: 3

Figure S1. PV (A, C) and TBARS (B, D) development during ice storage in minced mixed herring co-products following lab-scale pre-dipping in 2% Duralox MANC (A, B) or 0.2% rosemary extract (RE) with or without 0.5% isoascorbic acid (C, D). Fresh herring co-products were immersed in pre-chilled (4 °C) antioxidant-containing solutions for 30s in a 1:5 ratio (weight/volume) and then drained well (~15s) in a fine stainless steel strainer. The antioxidant-containing solutions were re-used up to 10 dipping cycles were done in the same solution. Data are shown as mean values ± standard deviation (SD) (n=2).

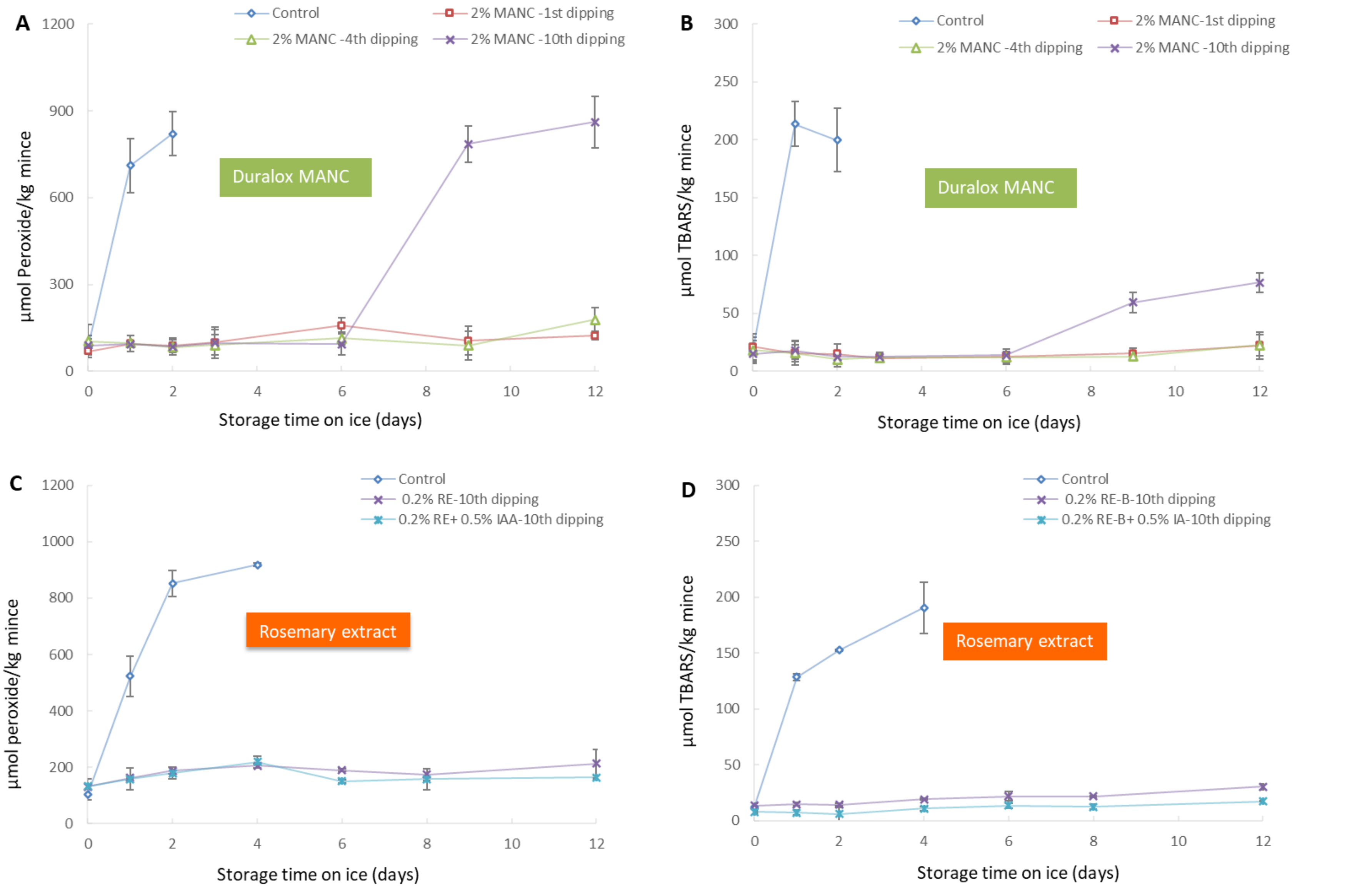

Figure S2. Details from the analysis of carnosol and carnosic acid by UPLC. Panel A and B show chromatograms; panel C and D show UV–visible spectrum; panel E and F show the standard curve for carnosol and carnosic acid, respectively.

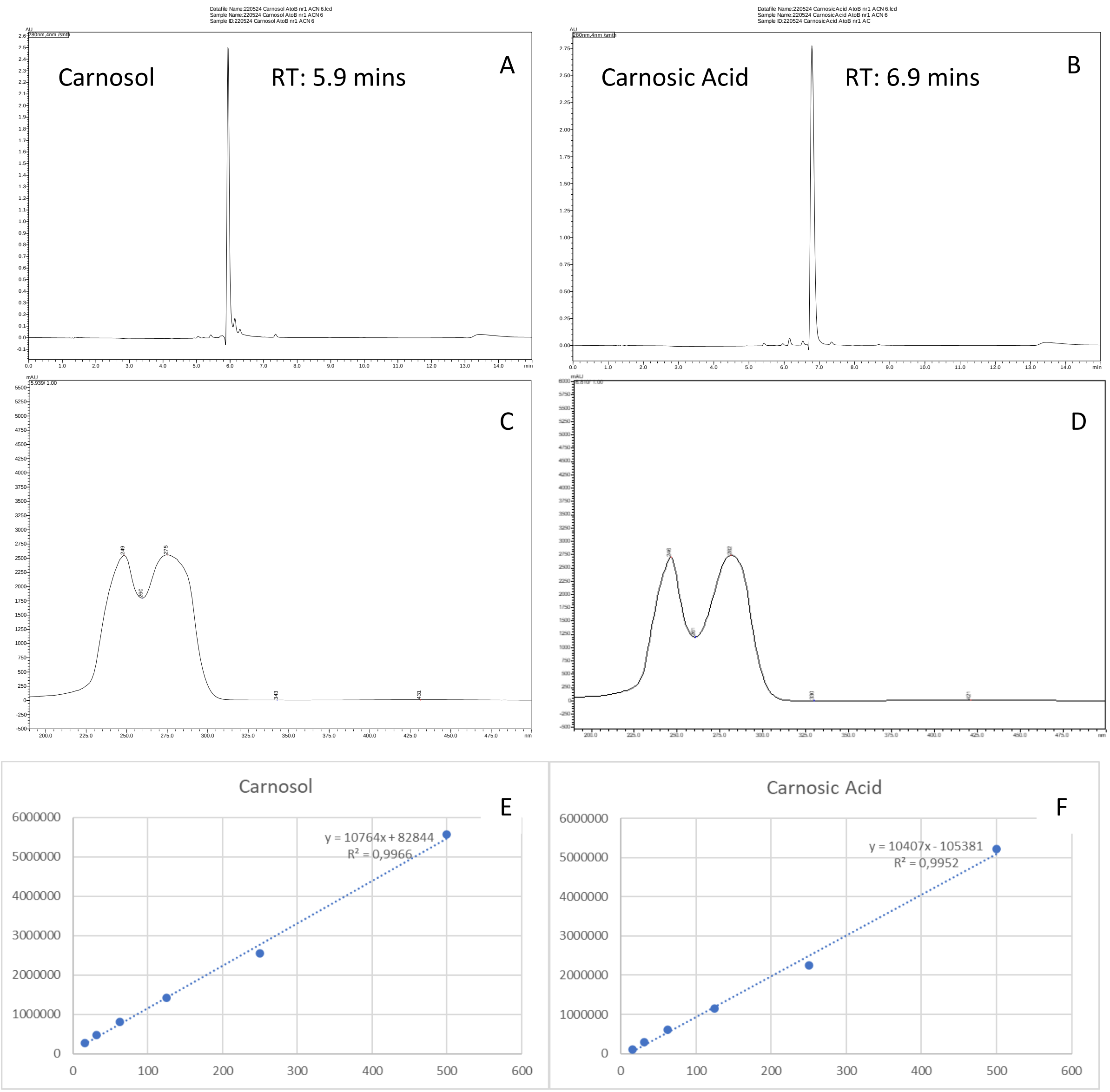

Figure S3. Carnosol (CN) and carnosic acid (CA) contents in the crude antioxidants used throughout the study; Duralox-MANC (A), rosemary extract (B). Data are shown as mean values ± standard deviation (SD) (n=2).

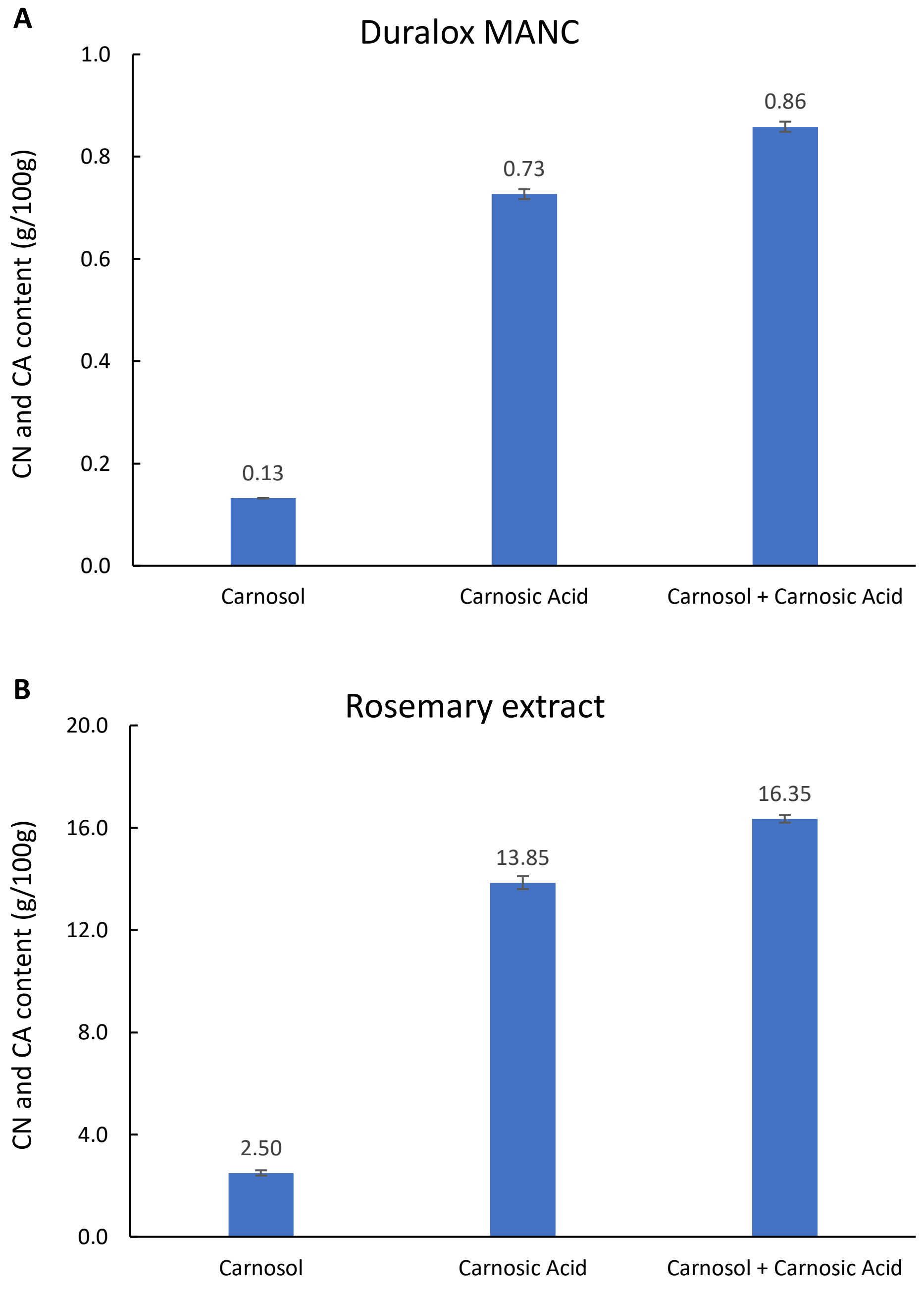

Supplement: Supplementary file 1 — sc2c07164_si_001.pdf [file sc2c07164_si_001.pdf]
